# The Preparation of Dental Alloys and Cements

**Published:** 1897-10

**Authors:** Louis Shaw

**Affiliations:** Brooklyn, N. Y.


### THE PREPARATION OF DENTAL ALLOYS AND CEMENTS.

BY LOUIS SHAW, M.D., BROOKLYN, N. Y.

In view of the recent investigations of Dr. Black and his papers on "Dental Alloys and Amalgams," I have thought that some simple and definite instructions for preparing alloys might be of value.

There are doubtless many who would like to use alloys made after formulæ suggested by these investigations, but not being familiar with the apparatus and methods necessary, think the preparation of a dental alloy more difficult than it is.

I shall not discuss the various ways in which an alloy can be made. Each manufacturer of an alloy no doubt has a way of combining the metals that he thinks best. But I shall describe a method that I have used for a number of years with satisfactory results.

For melting the metals I have used a gas-furnace made by the Buffalo Dental Manufacturing Company, and known as No. 40*a*,—Fletcher crucible furnace. This can also be had to work with kerosene. A plumbago crucible comes with the furnace. The other articles necessary are an ingot mould, a small pair of crucible tongs, and a short clay pipe-stem.

The silver is first melted, adding enough borax to cover its surface and protect it from the air. If the alloy is to contain copper, the blast must be kept up a few minutes to raise the temperature of the melted silver. The copper, in the form of very thin sheet or foil, is now added piece by piece.

If it is to be an alloy containing gold, the proper proportion of gold-foil scrap, wrapped in a piece of paper, is dropped into the crucible.

When the gold or copper is melted, the blast is kept on a few minutes and the other metals of the alloy added, those having the highest melting-points first. The tin, of course, will come last, and is best added in pieces the size of a large marble.

As soon as the last piece of tin is added the crucible is held in the furnace by tongs and its contents stirred vigorously with the clay pipe-stem, suitably fastened to a piece of wood. While still stirring, the metal is quickly poured into the ingot mould. The stirring and pouring before the metal comes to rest is to produce as uniform an ingot as possible.

Some may have difficulty in procuring pure metals. Pure silver, in grain form, is sold by gold and silver refiners. Pure copper, zinc, and tin can be bought from those who supply analytical and experimental chemists.

The ingot is readily reduced by a large coarse file, which must be kept for this purpose only. Passing a magnet through the thinly spread filings will remove any particles of steel from the file.

The filings must now be tempered as directed by Dr. Black, either by keeping them in an oven at a temperature of 120° F. for three days, or putting them in a glass flask and immersing the flask in boiling water for fifteen minutes.

Until I read Dr. Black's paper in the *Dental Cosmos* last August, I had been using two formulæ for alloys, based on Dr. Flagg's experiments. One was silver sixty parts, gold five parts, and tin thirty-five parts; the other substituted five parts of copper for the gold, leaving the silver and tin the same.

Lately I have adopted three formulæ suggested by Dr. Black. They are the following: one of silver and tin,—viz., silver, seventy-four parts; tin, twenty-six parts; one containing copper,—viz., silver, sixty-four parts; copper, four parts; zinc, one part; tin, thirty parts; and one containing gold,—viz., silver, sixty-eight and one-half parts; tin, twenty-five and one-half parts; gold, five parts; zinc, one part. Dr. Black speaks favorably of this last formula in the *Dental Cosmos* for December, 1896.

CEMENT.

As is generally known, cement powder is oxide of zinc, usually with some silica added with the idea of making it more resistant to wear.

In most of the directions for preparing this powder, oxide of zinc is dissolved in nitric acid, and the nitrate of zinc is afterwards heated to drive off the nitric acid, leaving zinc oxide.

This part of the process is enough to deter most dentists from trying to prepare cement powder, as the fumes of nitric acid are very corrosive, and difficult to get rid of, except through a chimney arranged for carrying off acid vapors.

This dissolving of the oxide is not necessary, and, by omitting it any one can prepare the powder with little difficulty. Most oxides of zinc made in the United States are too impure for dental use.

French oxide of zinc is much purer and makes a very good cement. Hubbuck's English oxide of zinc is the purest I have been able to obtain, and produces a very white cement. All other oxides I have tried produce a more or less yellow product. This yellow color is owing probably to traces of the oxides of other metals. These are not sufficient, however, in the oxides of zinc prepared for medicinal use, to affect their value for cement. If a white cement is not desired, they answer very well.

The oxide is placed in a sand crucible and the cover luted on with potters' clay mixed with water.

The crucible is now placed in a coal-fire—a range will do—and covered with coal, so that it will all be brought to a red heat. After being held at a red heat for two hours, it is removed and allowed to cool.

The oxide is now removed, and rubbed to a fine powder in a Wedgewood mortar, when it is bottled to keep it from the air.

The liquid is made by dissolving in water sufficient glacial phosphoric acid to make a dense syrupy solution.

It is difficult to state the exact composition of the liquid chemically, as all commercial glacial phosphoric acid contains from seven to fourteen per cent. of sodium phosphate. On being dissolved, the glacial phosphoric acid slowly takes up another equivalent of water, and finally a third, becoming at last ortho-phosphoric acid. The liquid may then be a mixture of the three phosphoric acids holding sodium phosphate in solution. My knowledge of cement liquid is unsatisfactory, and I cannot always get uniform results with different lots. I have not been able to get pure glacial phosphoric acid, but hope with further study of the phosphoric acids to be able to prepare a liquid that will be always uniform. The process described, however, produces an oxyphosphate cement that compares favorably with any I have purchased, both as to working qualities and insolubility.

